# Non-invasive visualisation of long-lasting brain metabolic alterations in murine pseudo-infection model using parahydrogen-polarised [1-^13^C] pyruvate MRI

**DOI:** 10.1186/s11671-025-04304-9

**Published:** 2025-08-06

**Authors:** Hayate Tomiyama, Masaki Yamasaki, Takayuki Isagawa, Norihiko Takeda, Takuya Hashimoto, Hiroshi Hirata, Shingo Matsumoto

**Affiliations:** 1 Division of Bioengineering and Bioinformatics, Graduate School of Information Science and Technology, Hokkaido University, Sapporo, 060-0814 Japan; 2 Department of Electronics and Information Engineering, School of Engineering, Hokkaido University, Sapporo, 060-0814 Japan; 3 Division of Cardiology and Metabolism, Center for Molecular Medicine, Jichi Medical University, Shimotsuke, 329-0498 Tochigi Japan; 4 Department of Cardiovascular Medicine, Graduate School of Medicine, The University of Tokyo, Tokyo, 113-8655 Japan; 5 Molecular Synthesis and Function Laboratory, RIKEN Cluster for Pioneering Research, Wako, 351-0198 Saitama Japan; 6 Division of Bioengineering and Bioinformatics, Faculty of Information Science and Technology, Hokkaido University, North 14, West 9, Kita-ku, Sapporo, 060-0814 Hokkaido Japan

**Keywords:** Brain metabolism, Infection, Immune fatigue, Hyperpolarised ^13^C MRI, PHIP, Quantum sensing

## Abstract

Long-lasting neurological issues, including cognitive impairment, anxiety, and depression, that persist after recovery from acute inflammatory diseases, such as infections, have become a significant social problem, particularly following the coronavirus disease 2019 pandemic. Various diagnostic techniques and biomarkers have been explored to objectively evaluate brain symptoms associated with infection–induced local or systemic inflammatory responses (i.e. immune fatigue); however, their detection capabilities remain limited. Here we investigated whether magnetic resonance imaging (MRI) combined with a quantum-sensed molecule, parahydrogen-polarised [1-^13^C] pyruvate, could detect persistent brain metabolic alterations in a murine pseudo-infection model induced by polyinosinic–polycytidylic acid (Poly(I: C)), a Toll-like receptor 3 ligand. Significant alterations in brain pyruvate metabolism favouring glycolysis were observed in both the acute and late phases of the pseudo-infection model, with a 12.7% and 2.5% decrease in bicarbonate flux, and a 58.4% and 32.2% increase in lactate flux on day 3 and week 2, respectively. These brain metabolic changes were accompanied by diminished dopamine signal markers in the striatum and nigra/ventral tegmental areas and reduced spontaneous nocturnal locomotor activity. A biochemical analysis of energy metabolic markers consistently supported the reprogramming of brain glucose metabolism, showing the suppression of oxidative phosphorylation during the acute phase and promotion of glycolysis during the late phase of Poly(I: C) treatment. Hyperpolarised ^13^C MRI of pyruvate metabolism is a promising non-invasive imaging biomarker for brain issues during the late phase of systemic infections and other neurodegenerative and psychiatric diseases, particularly in conditions lacking discernible morphological abnormalities.

## Introduction

Since December 2019, long-lasting symptoms that persist well beyond the acute phase of coronavirus disease 2019 (COVID-19) or emerge after the acute disease phase, known as post-COVID-19 syndrome or long COVID, have become a worldwide problem [1]. A recent meta-analysis revealed the presence of post-COVID-19 syndrome in 30% of patients even 2 years after COVID-19 infection, with fatigue, cognitive disorders, and pain being the most prevalent symptoms [2]. Despite advances in the treatment and mitigation of critical illnesses caused by infections, millions of survivors still have devastating neurological, cognitive, and psychiatric sequelae of COVID-19 infection [3]. The evaluation of persistent neurological symptoms including depression, cognitive impairment, anxiety, and sleep disorders following infection–induced local or systemic inflammatory responses, i.e., ‘immune fatigue’, presents a significant diagnostic challenge for which only limited diagnostic methods have been established. Traditional methods may not provide sufficient insight into the underlying mechanisms responsible for these persistent neurological symptoms, necessitating the development of advanced non-invasive imaging techniques to enable assessment objectively.

Diagnosing post-infection neurological symptoms using non-invasive imaging techniques is difficult because of their non-specificity and high variability which may overlap with other pathologic conditions. Conventional neuroimaging methods, such as magnetic resonance imaging (MRI) and computed tomography, provide structural insight and are useful for diagnosing brain issues with neurological degeneration; however, they frequently miss the finer functional and metabolic abnormalities associated with post-infectious/inflammatory brain dysfunction. Positron emission tomography (PET) with [^18^F] fluorodeoxyglucose ([^18^F] FDG), which measures glucose metabolic activity in the brain, can be used to identify areas of hypometabolism correlated with cognitive dysfunction. A [^18^F] FDG PET study of patients with long COVID symptoms revealed pathological results in 10/15 patients with predominant frontoparietal hypometabolism, and a voxel-wise principal component analysis of [^18^F] FDG uptake showed a high correlation with the Montreal Cognitive Assessment performance, whereas cerebral perfusion MRI did not reveal significant differences compared with a control group [4]. While PET scans have been useful for understanding the metabolic impact of neuroinflammation and the accompanying immune responses to infections in the brain, the need for radioactive tracers limits the practicality of routine diagnosis.

Hyperpolarised ^13^C MRI is an alternative quantum imaging technique in which the MRI signal of quantum-sensed stable isotope ^13^C is transiently enhanced more than 10,000 times, allowing for the real-time visualization of metabolic reactions in the body by MRI without the risk of ionising radiation exposure [5, 6]. Hyperpolarised [1-^13^C] pyruvate may be among the most useful hyperpolarised ^13^C-labelled tracers for visualising mitochondrial energy metabolism [7–10]. Pyruvate, a key branching point in glucose metabolism, is divided into the tricarboxylic acid (TCA) cycle for oxidative phosphorylation, glycolysis, and amino acid synthesis [5, 11, 12]. Using the hyperpolarised [1-^13^C] pyruvate MRI, we non-invasively visualised the metabolic reprogramming, increased the metabolic flux through pyruvate-to-lactate and pyruvate-to-alanine, in the liver of a pseudo-infection model during the acute phase [13].

In the brain, there is accumulating evidence for a unique metabolic cooperation mechanism between astrocytes and neurones in the transport of lactate, a byproduct of glycolysis, known as the astrocyte–neuron lactate shuttle (ANLS) [14, 15]. Astrocytes, which wrap around blood vessels in the cerebral parenchyma, take up glucose from the bloodstream, metabolise it to lactate via the glycolytic pathway, and export it to the extracellular milieu. Lactate can be captured by neurones through monocarboxylate transporter 2, converted back to pyruvate, and utilised in the TCA cycle for mitochondrial adenosine triphosphate (ATP) production. A recent study of hyperpolarised [1-^13^C] pyruvate MRI revealed that RF saturation of hyperpolarised [1-^13^C] lactate significantly reduces the detectable [^13^C] bicarbonate signal due to possible intracellular and intercellular lactate–bicarbonate exchange in the rat brain, likely associated with the ANLS [16]. The pyruvate-to-lactate metabolic flux can be altered in cases of neuroinflammation due to pro- or anti-inflammatory responses. In neurodegenerative diseases, the activated microglia undergo metabolic reprogramming from oxidative phosphorylation to aerobic glycolysis, leading to increased lactate production. In turn, lactate promotes the release of pro-inflammatory cytokines, leading to chronic neuroinflammation [17]. On the other hand, in the anti-inflammatory response by lactate, because the use of fatty acids as hydrogen donors is accompanied by severe β-oxidation-associated reactive oxygen spices (ROS) generation that impairs neurotransmitters release [18], neurones spurn hydrogen-rich fatty acids for oxidative ATP synthesis [19]. However, under stress conditions, hyperactivated neurones use fatty acids to produce energy.

To protect neurones from excess ROS generation during periods of enhanced activity, astrocytes promote glycolytic metabolism to provide lactate as an energy substrate. Unused fatty acids in neurons are transferred as lipid droplets to antioxidant-rich astrocytes and used as fuel for mitochondrial oxidative phosphorylation [20, 21]. Therefore, brain lactate concentration or the metabolic flux shift from oxidative phosphorylation to lactate production can be key biomarkers of neurological pathophysiology. In fact, hippocampal lactate concentration decreases in physiological stress models but increases under psychological stress [22]. A large-scale animal model study of 109 strains/conditions also revealed a strong association between brain lactate levels and behavioural abnormalities involving cognitive impairment [23]. Collectively, the feasibility of hyperpolarised ^13^C MRI for non-invasive visualization of pyruvate-to-lactate metabolic flux, in addition to bicarbonate as a metabolic flux for oxidative phosphorylation, can be a unique imaging biomarker for monitoring the persistent neurological disorders after immune fatigue, neuroinflammation, and infection.

In this study, we investigated whether the hyperpolarised ^13^C MRI of pyruvate metabolism can detect persistent brain metabolic alterations lasting beyond the acute phase in a murine polyinosinic–polycytidylic acid (Poly(I: C))–induced immune fatigue/pseudo-infection model, and its association with neurological activity markers and behavioural abnormalities. Poly(I: C) is a Toll–like receptor 3 (TLR3) agonist that has been reported to induce systemic immune responses, cause neuroinflammation, and impair dopaminergic neurons, thereby serving as a mimic of RNA virus infection.

## Methods

### Preparation of pseudo-infection model mice

All animal experiments were performed in accordance with the Law for the Care and Welfare of Animals in Japan and approved by the Animal Experiment Committee of Hokkaido University (approval no. 21-0007). Female C3H/HeYorkSlc mice were purchased from Japan SLC (Hamamatsu, Japan). All mice were housed in cages containing 2–5 individuals and provided with food and water *ad libitum*. The mice were maintained on a 12:12 light-dark cycle. The pseudo-infection (immune fatigue) model was established by an intraperitoneal injection of Poly(I: C), a Toll-like receptor 3 (TLR3) ligand, at a dose of 100 µg/day (approximately 5 mg/kg body weight) for 3 consecutive days. The systemic immune response was confirmed by monitoring the blood levels of inflammatory cytokines, including tumour necrosis factor-α (TNF-α), interleukin-6 (IL-6), and interleukin-1β (IL-1β), using enzyme-linked immunosorbent assay kits (Proteintech KE10002, KE10007, and KE10003, respectively).

To avoid potential confounding effects of hyperpolarised ^13^C MRI measurements—including exogenous pyruvate injection and stress induced by isoflurane anesthesia, physical restraint, and tail vein cannulation—on subsequent neurological, biochemical, and behavioral assessments, all experiments, including those performed at different time points, were conducted using independent groups of mice.

### Evaluation of spontaneous behavioural abnormalities

The mice were placed in a polycarbonate cage (30 cm W × 19 cm D × 13 cm H), and their nocturnal movements were recorded and analysed using lab-made software written in Python connected to an overhead video camera. Water bottle and feed were placed diagonally within each cage. Each trial was conducted for 11 h (7:00 p.m. to 6:00 a.m.), and the total distance travelled per hour was analysed.

### Biochemical and neurochemical analysis of murine brain

#### Immunohistochemical analysis

Brain tissues were fixed in 4% paraformaldehyde, and 4-µm-thick paraffine sections were prepared. After the application of antigen retrieval citrate buffer at 95 °C for 20 min, the sections were incubated with primary antibodies overnight at 4 °C, followed by incubation with a horseradish peroxidase–conjugated secondary antibody for 1 h at room temperature. The signal was developed using 3,3′-diaminobenzidine. The following primary antibodies and dilutions were used: rabbit anti-LDH-A (NBP1-48336, Novus Biologicals), 1:1000 and anti-tyrosine hydroxylase (TH) (1:2000; ab137869; Abcam).

#### Brain and lactate concentrations

Brain homogenates (1:10 w/v) were centrifuged at 12,000 × *g* for 5 min at 4 °C, and the supernatant was analysed. The lactate concentration was determined using a Lactate Assay Kit-WST (L256; Dojindo Lab) in accordance with the manufacturer’s instructions.

#### Simple western analysis

Brain tissue (150–200 mg) was homogenised in 20 volumes of protein extraction reagent (T-PER™; Thermo Fisher Scientific Inc.) containing protease inhibitors (Halt™ Protease Inhibitor Cocktail; Thermo Fisher Scientific Inc.), centrifuged (12000 × *g* for 5 min at 4 °C), and the supernatant was stored at − 80 °C. Simple western analysis was performed using an automated system (Abby, ProteinSimple Corp.). The following primary antibodies were used: rabbit anti-GAPDH (14C10) (1:4000; 2118; Cell Signalling Technology); and anti-phospho-PDH (1:150; 37115; Cell Signalling Technology).

### Hyperpolarised ^13^C MRI study of brain metabolic alterations

#### Parahydrogen–induced polarisation of [1-^13^C] pyruvate

A hyperpolarised [1-^13^C] pyruvate solution was prepared using a previously reported parahydrogen–induced polarisation (PHIP) system [6]. Briefly, [1-^13^C] propargylpyruvate (20 µL) and hydrogenation catalyst [Rh(dppb)(COD)]BF_4_ (20 mg; #341134; Sigma-Aldrich) were dissolved in 1 mL of chloroform. The system automatically; (i) performed a parahydrogenation reaction at 55–60 °C for 10 s under 0.60 MPa of parahydrogen gas; (ii) facilitated ^1^-^13^C spin order transfer; (iii) mixed the solution with 0.9 mL of hydrolysis solution containing 62.5 mM NaOD, 50 mM Tris-HCl, and 0.5 mM EDTA-2Na in D_2_O; and (iv) bubbled nitrogen gas at 75 °C for 23 s to vaporise chloroform and allylalcohol generated by hydrolysis (The catalyst is precipitated during this procedure). This produced ~ 0.6 mL of aqueous solution containing 80–90 mM hyperpolarised [1-^13^C] pyruvate (pH 7.0–8.5) with ^13^C polarisation of 3–5% at the time of MRI measurements.

#### Chemical shift imaging of hyperpolarised [1-^13^C] pyruvate metabolism in brain

In vivo hyperpolarised ^13^C MRI of brain pyruvate metabolism was conducted using a 1.5 T lab-made MRI system with a Japan REDOX spectrometer and RF coils (inner solenoid for ^13^C, outer saddle for ^1^H). After T_2_-weighted fast-spin echo imaging, hyperpolarised [1-^13^C] pyruvate (80–90 mM; 10 µL/g body weight), following a commonly used injection protocol in both d-DNP and PHIP mouse studies [24], was injected through the tail vein over the course of 12 s. After waiting 13 s for tracer distribution to the brain, two-dimensional spatially phase-encoded ^13^C chemical shift imaging (CSI) with centric k-space acquisition (CSI acquisition time = 20 s). The scan parameters were as follows: 16 × 16 matrix; field of view = 32 × 32 mm; slice thickness = 12 mm; echo time/repetition time = 10/75 ms; flip angle = 10°; and spectral bandwidth = 2 kHz (122.4 ppm) for 128 points. To denoise the CSI image data, rank-reduced image matrices were generated using tensor decomposition with a small core-tensor size [25]. The size of the core-tensor, which corresponds to the rank of the CSI image matrices, was set to 8 and 12 for each spatial and spectral dimension, respectively. The CSI data (16 × 16 matrix size) were processed using an anatomical ^1^H MRI-guided super-resolution technique [26], in which corresponding T_2_-weighted ^1^H MRI slices, down-sampled from a 128 × 128 to 64 × 64 matrix, were used as structural guide images and reconstructed into a 64 × 64 matrix. After baseline correction, the area under the curve (AUC) of each metabolite peak was calculated over the whole brain region. The resulting brain images were displayed as metabolite ratio maps using brain ROI masks selected from the corresponding T_2_-weighted ^1^H MRI images. The overall MRI scan time per mouse was 30–40 min, including anatomical ^1^H MRI scans and the preparation time for the hyperpolarised [1-^13^ C] pyruvate solution.

### Statistical analysis

An unpaired, two-sided Student’s t-test was used to compare each treatment group with the pre-treatment control group. Quantitative data are presented as mean ± standard deviation (SD) for each group.

## Results and discussion

### Systemic inflammatory response and sustained abnormality of spontaneous activity in pseudo-infection model mice

**Fig. 1:**
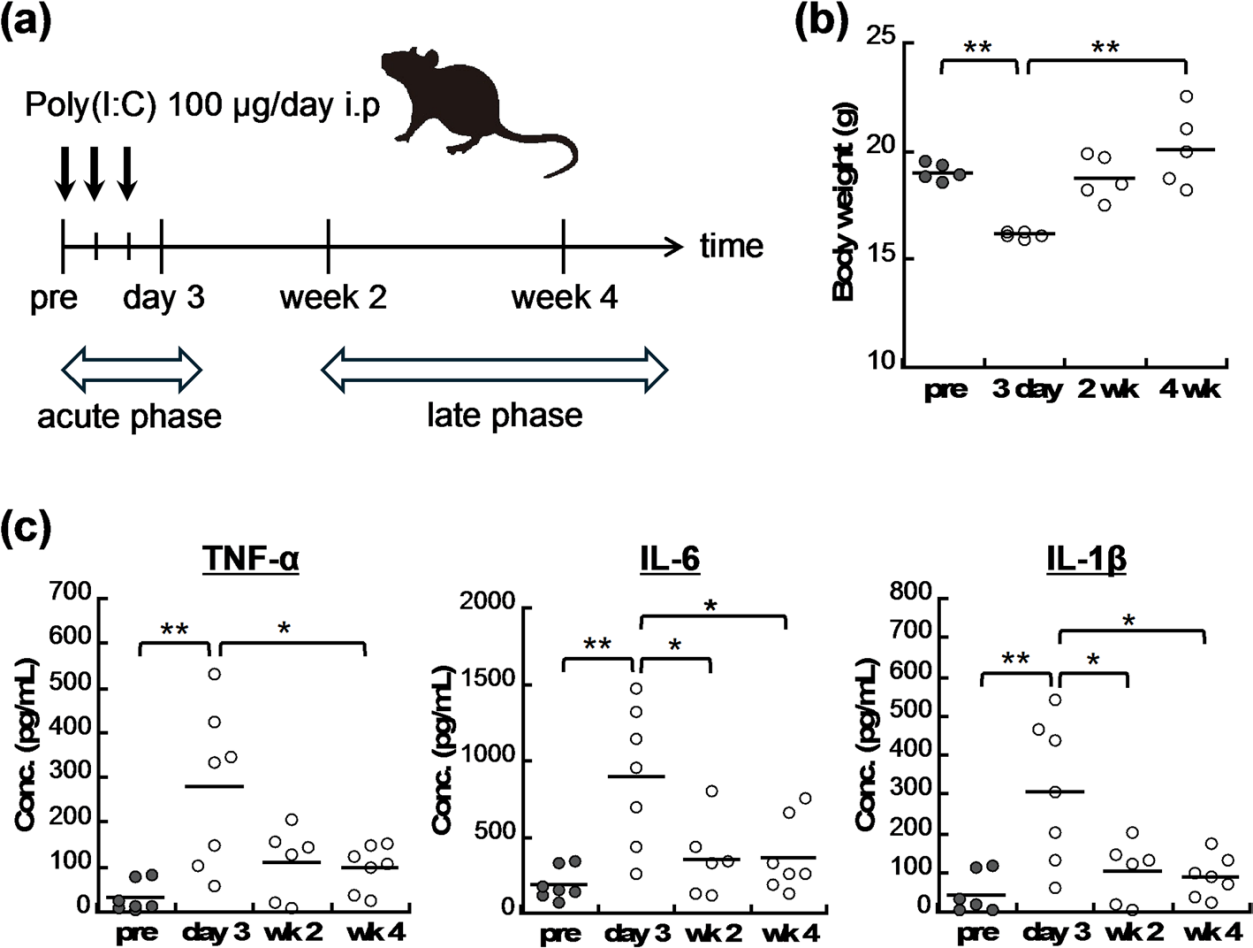
Systemic inflammatory responses in Poly(I: C)–induced pseudo-infection model mice. **a** Experimental plan for the generation and evaluation of Poly(I: C)–induced pseudo-infection model mice. **b** Body weight significantly decreased 3 days after the initial intraperitoneal Poly(I: C) administration and returned to pre-injection levels within 2 weeks. **c** Plasma concentrations of inflammatory cytokines—TNF-α, IL-6, and IL-1β—significantly increased 3 days after the initial Poly(I: C) administration and recovered to pre-injection level within 2 weeks. *n* = 5–7, *p < 0.05, **p < 0.01. IL-1β, interleukin-1β; IL-6, interleukin-6; Poly(I: C), polyinosinic–polycytidylic acid; TNF-α, tumour necrosis factor-α

Long-lasting effects of RNA viral infections on brain function can be induced by the binding of a virus to TLR3 in the liver, resulting in a systemic inflammatory response (i.e. immune fatigue) in addition to its direct binding to brain TLR3 [27]. Poly(I: C) is an artificially synthesised RNA that binds to both brain and liver TLR3 and mimics the symptoms observed in RNA virus infections. After the continuous administration of Poly(I: C) for 3 days, mouse body weight was significantly decreased (Fig. 1). The systemic inflammatory response was confirmed via monitoring of the blood levels of inflammatory cytokines. The concentrations of TNF-α, IL-6, and IL-1β significantly increased after 3-day Poly(I: C) administration. Both body weight and circulating inflammatory cytokine concentrations returned to pre-treatment levels within 2 weeks after Poly(I: C) treatment.

**Fig. 2:**
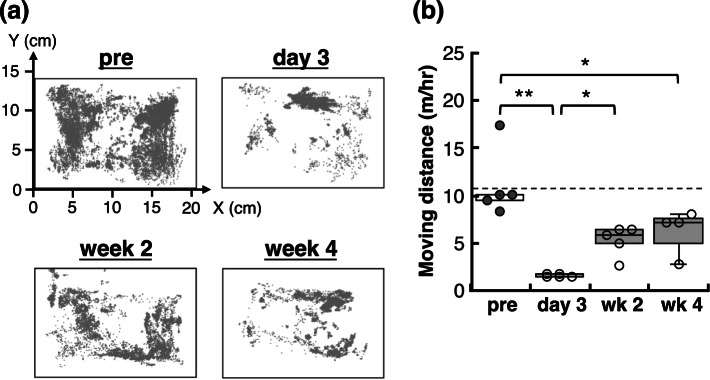
Diminished spontaneous nocturnal locomotor activity in pseudo-infection model mice. **a** Locations of representative mice in cages at night before and 3 days, 2 weeks, and 4 weeks after the initial Poly(I: C) administration recorded by an overhead infrared camera. **b** Locomotor activity analysis revealed a significant decreased moving distance at night-time in the mice 3 days after the initial Poly(I: C) administration, which did not return to pre-injection within 4 weeks (moving distance: pretreatment: 11.07 ± 3.23 m/hr, day 3: 1.59 ± 0.13 m/hr, week 2: 5.24 ± 1.42 m/hr, week 4: 5.24 ± 1.42 m/hr). *n* = 4–5, *p < 0.05, **p < 0.01

The spontaneous locomotor activity of the mice was evaluated by monitoring of their behaviour using an overhead camera overnight (7:00 p.m. to 6:00 a.m.). The nocturnal movement distance per hour significantly decreased after 3 days of Poly(I: C) treatment and gradually increased thereafter but never returned to pre-treatment levels even 4 weeks later (Fig. 2). This suggests that the effects of Poly(I: C) treatment persisted in the brains of the mice even after the systemic inflammation had completely subsided. Based on these observations of the systemic inflammatory response and behavioural abnormalities, we conducted the following experiments during the acute phase (day 3) and the late phase (weeks 2 and 4).

### Non-invasive imaging of brain metabolic alterations using parahydrogen-polarised ^13^C MRI of pyruvate in the pseudo-infection model mice

**Fig. 3:**
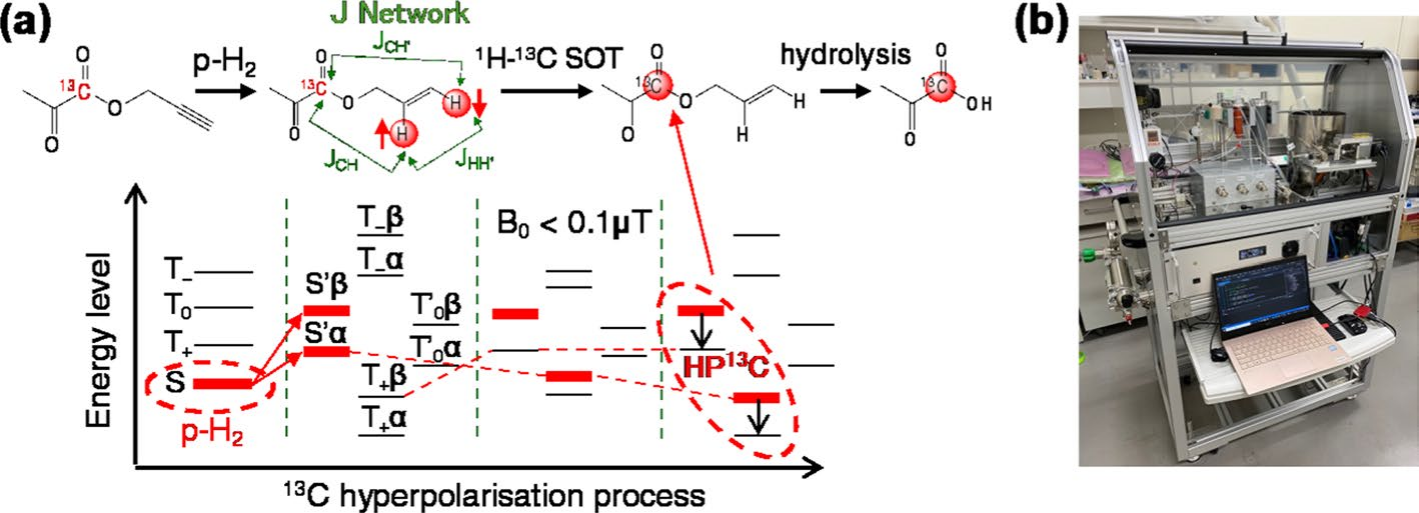
Parahydrogen–induced polarisation of [1-^13^C] pyruvate. **a** Schematic illustration of the preparation of hyperpolarised [1-^13^C] pyruvate using the PHIP sidearm hydrogenation technique. **b** A photo of the automated PHIP polariser system

Hyperpolarised ^13^C MRI is a rapidly advancing technology that transiently enhances the MRI signal of ^13^C-labelled tracers by more than 10,000 times, enabling non-invasive visualisation of their metabolic reactions. Using hyperpolarised ^13^C MRI, we successfully observed metabolic reprogramming in the livers of Poly(I: C)-treated mice during the acute phase [13]. Although hyperpolarised ^13^C MRI has been extended to brain metabolic imaging, its applications to date have been limited to brain diseases such as brain tumours, traumatic brain injuries, and multiple sclerosis [28–31]. Its feasibility for detecting metabolic alterations in psychological disorders or neurodegenerative diseases without discernible morphological abnormalities remains unclear. In this study, instead of using conventional dissolution dynamic nuclear polarisation (d-DNP)–based polariser systems, we employed the PHIP sidearm hydrogenation technique (Fig. 3a) [32, 33] in our recently developed PHIP polariser system (Fig. 3b) [6, 29] to produce hyperpolarised [1-^13^C] pyruvate. We then investigated whether hyperpolarised [1-^13^C] pyruvate MRI could detect brain metabolic alterations associated with behavioural abnormalities in the pseudo-infection model.

**Fig. 4:**
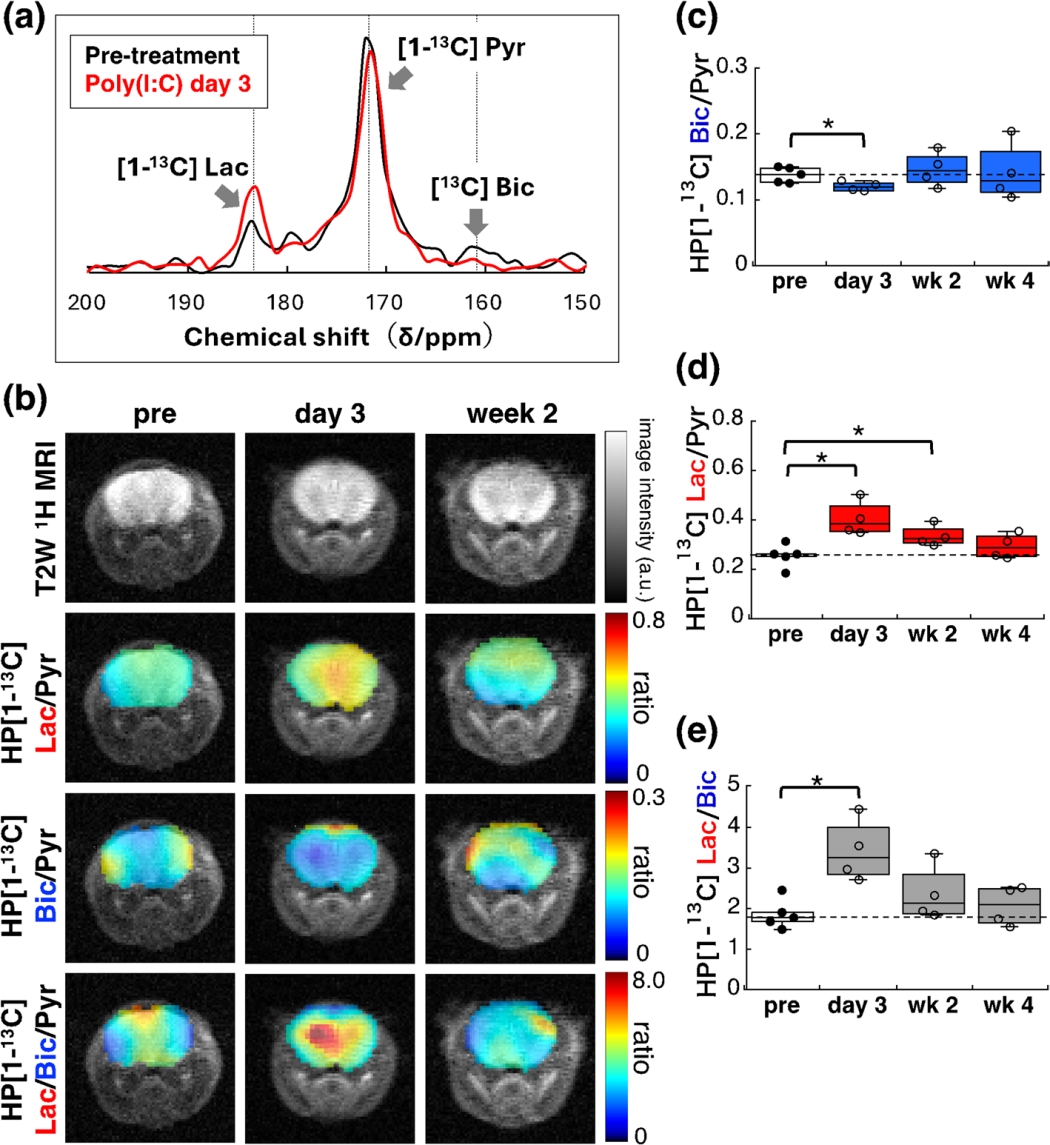
Hyperpolarised ^13^C MRI of brain pyruvate metabolism in the pseudo-infection model. **a** Representative ^13^C MR spectrum in the brain acquired by CSI after injection of hyperpolarised [1-^13^C] pyruvate. **b** Representative images of T_2_-weighted ^1^H MRI, hyperpolarised [1-^13^C] lactate-to-pyruvate (Lac/Pyr), bicarbonate-to-pyruvate (Bic/Pyr), and lactate-to-bicarbonate (Lac/Bic) ratios in brains of mice before, 3 days, and 2 weeks after Poly(I: C) administration. **c** Brain metabolic flux of hyperpolarised [1-^13^C] pyruvate into bicarbonate, which reflects the metabolic flux to oxidative phosphorylation, was significantly reduced in mice 3 days after Poly(I: C) treatment compared to pre-treatment levels. **d** The brain metabolic flux of pyruvate into lactate was also significantly increased in mice 3 days and 2 weeks later Poly(I: C) administration. **e** The brain metabolic flux ratio of hyperpolarised [1-^13^C] lactate-to-bicarbonate (Lac/Bic), representing the glycolysis-to-oxidative phosphorylation flux ratio, was significantly increased 3 days after Poly(I: C) administration. *n* = 4–5, *p < 0.05

Representative ^13^C MR spectrum and parametric images of hyperpolarised ^13^C lactate-to-pyruvate (Lac/Pyr), bicarbonate-to-pyruvate (Bic/Pyr), and lactate-to-bicarbonate (Lac/Bic) flux ratios in the brain taken before, 3 days into administration, and 2 weeks after Poly(I: C) administration, are shown in Fig. 4a and b. During the acute phase on day 3, a significant suppression of pyruvate metabolic flux to oxidative phosphorylation (Bic/Pyr: pre-treatment: 0.138 ± 0.011; day 3: 0.121 ± 0.006, *p* < 0.05, − 12.7% vs. pre-treatment; week 2: 0.147 ± 0.023, −.5% vs. pre-treatment; week 4: 0.142 ± 0.038, + 3.1% vs. pre-treatment) and a simultaneous increase in metabolic flux to lactate (Lac/Pyr: pre-treatment: 0.256 ± 0.041; day 3: 0.406 ± 0.061, *p* < 0.05, + 58.4% vs. pre-treatment; week 2: 0.336 ± 0.038, *p* < 0.05, + 32.2% vs. pre-treatment; week 4: 0.295 ± 0.043, + 14.9% vs. pre-treatment) were observed (Fig. 4c and d). The significant increase in the Lac/Pyr flux ratio persisted in the late phase (2 weeks after Poly(I: C) treatment). Similarly, the ratio of hyperpolarised ^13^C Lac/Bic, which indicates a metabolic flux balance between oxidative phosphorylation and glycolysis, was significantly elevated during the acute phase (Lac/Bic: pre-treatment: 1.86 ± 0.33; day 3: 3.40 ± 0.066, *p* < 0.05, + 82.4% vs. pre-treatment; week 2: 2.36 ± 0.60, + 40.3% vs. pre-treatment; week 4: 2.16 ± 0.43, + 15.8% vs. pre-treatment) (Fig. 4e). The Lac/Pyr and Lac/Bic ratios gradually decreased during the late phase but did not return to pre-treatment levels, even 4 weeks after Poly(I: C) treatment. These results suggest that hyperpolarised [1-^13^C] pyruvate MRI can non-invasively capture long-lasting metabolic alterations in the brains of Poly(I: C)–induced pseudo-infection model mice.

In the pseudo-infection model mice, the metabolic flux to hyperpolarised ^13^C bicarbonate can serve as a promising key indicator of neurological dysfunction. However, detection of hyperpolarised ^13^C bicarbonate in the rodent brain is frequently reported to be challenging. There are at least three factors that may contribute to this difficulty. First, Bøgh et al. clearly demonstrated that RF saturation of the hyperpolarised [1-^13^C] lactate peak suppresses the apparent metabolic flux from injected hyperpolarised ^13^C pyruvate to bicarbonate in the rat brain [16]. This implies a contribution of ANLS, in which pyruvate is metabolized to lactate in astrocytes. The lactate is then secreted, taken up by neurons, converted back to pyruvate, and used for oxidative phosphorylation. This process results in carbon dioxide production in neurons, which is subsequently converted into bicarbonate by carbonic anhydrase. Thus, unlike in most other organs where pyruvate-to-bicarbonate conversion involves a two-step process, in the brain this involves multiple steps, making detection difficult due to the short lifetime of the hyperpolarised ^13^C signal. Second, unlike in human studies, preclinical studies typically require the use of anesthesia during MRI scans, which suppresses both hemodynamics and cerebral metabolism [34]. Since ANLS is coupled to glutamatergic neuronal activity, neuronal activation is essential for lactate shuttling from astrocytes to neurons and subsequent conversion to bicarbonate. To minimize the suppressive effect of isoflurane on brain metabolism, we first performed anatomical ^1^H MRI scans under 1.5–2.0% isoflurane with a respiratory rate of 40–60 breaths per minute. Then, prior to hyperpolarised ^13^C imaging, we reduced isoflurane to 0.5–1.0% and confirmed a respiratory rate of 70–100 breaths per minute. Third, most hyperpolarised ^13^C pyruvate MRI studies reporting difficulty in detecting brain ^13^C bicarbonate have been performed at high magnetic fields over 7T [31], whereas successful detection has almost exclusively been reported at lower field strengths (e.g., 3T) including this study at 1.5T [16, 28]. This may be attributed to anisotropic relaxation effects on the ^13^C–labeled carbonyl carbon, which lead to significantly faster hyperpolarised ^13^C signal decay at higher fields. As a substantial portion of bicarbonate production from injected ^13^C-pyruvate in the brain proceeds through this multi-step, intercellular pathway, the shortened lifetime of the hyperpolarised ^13^C signal due to carbonyl carbon anisotropy further complicates detection of hyperpolarised ^13^C bicarbonate at higher field strengths.

### Diminished brain dopamine synthesis

**Fig. 5:**
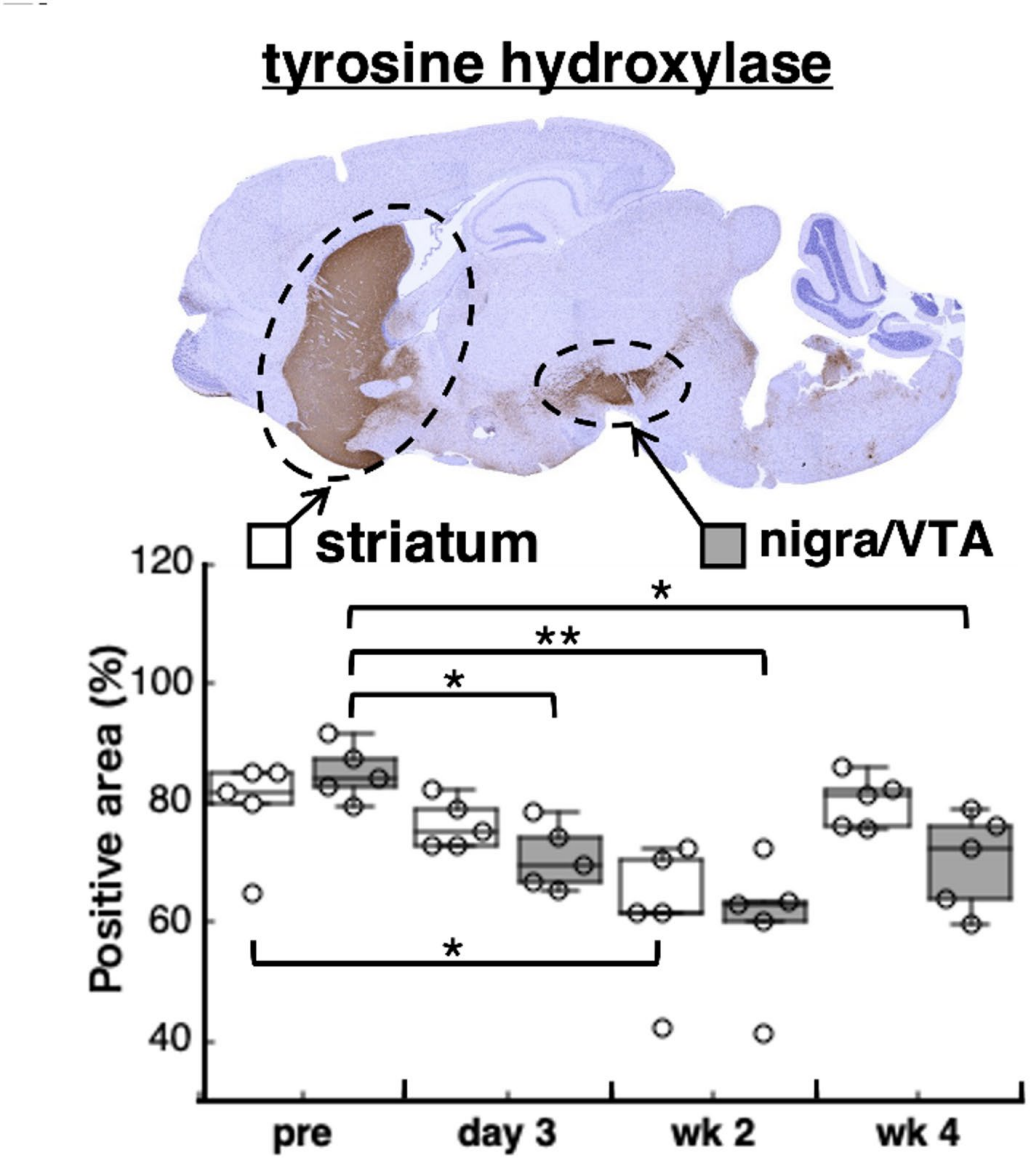
Attenuation of brain dopamine synthesis in the pseudo-infection mouse model. Immunohistochemical analysis showed that expression levels of tyrosine hydroxylase, a late-limiting enzyme of dopamine synthesis, in striatum and nigra/VTA gradually decreased after Poly(I: C) administration, with the most significant observed at 2 weeks. *n* = 5, *p < 0.05, **p < 0.01. VTA, ventral tegmental area

Alterations in systemic and neurological dopamine metabolism may contribute to the development of behavioural and psychiatric abnormalities associated with infections including COVID-19 and influenza [35, 36]. The viral mimic Poly(I: C) is also known to reduce striatal dopamine release by promoting ROS generation from the surrounding microglia [18]. An immunohistochemical analysis of TH, a rate-limiting enzyme in dopamine synthesis, was performed to investigate the long-term effects of Poly(I: C)–induced neuroinflammation on dopamine synthesis. The expression of TH in the striatum and nigra/ventral tegmental area (VTA) gradually decreased after Poly(I: C) administration (Fig. 5). The most significant reduction was observed at 2 weeks in both regions, and a significant reduction in TH expression was sustained for 4 weeks in the nigra/VTA after Poly(I: C) treatment. This long-lasting alteration in the dopamine synthesis marker may at least partially explain the spontaneous behavioural abnormalities observed during the late phase of Poly(I: C) treatment (Fig. 2).

### Alterations in brain energy metabolic markers

**Fig. 6:**
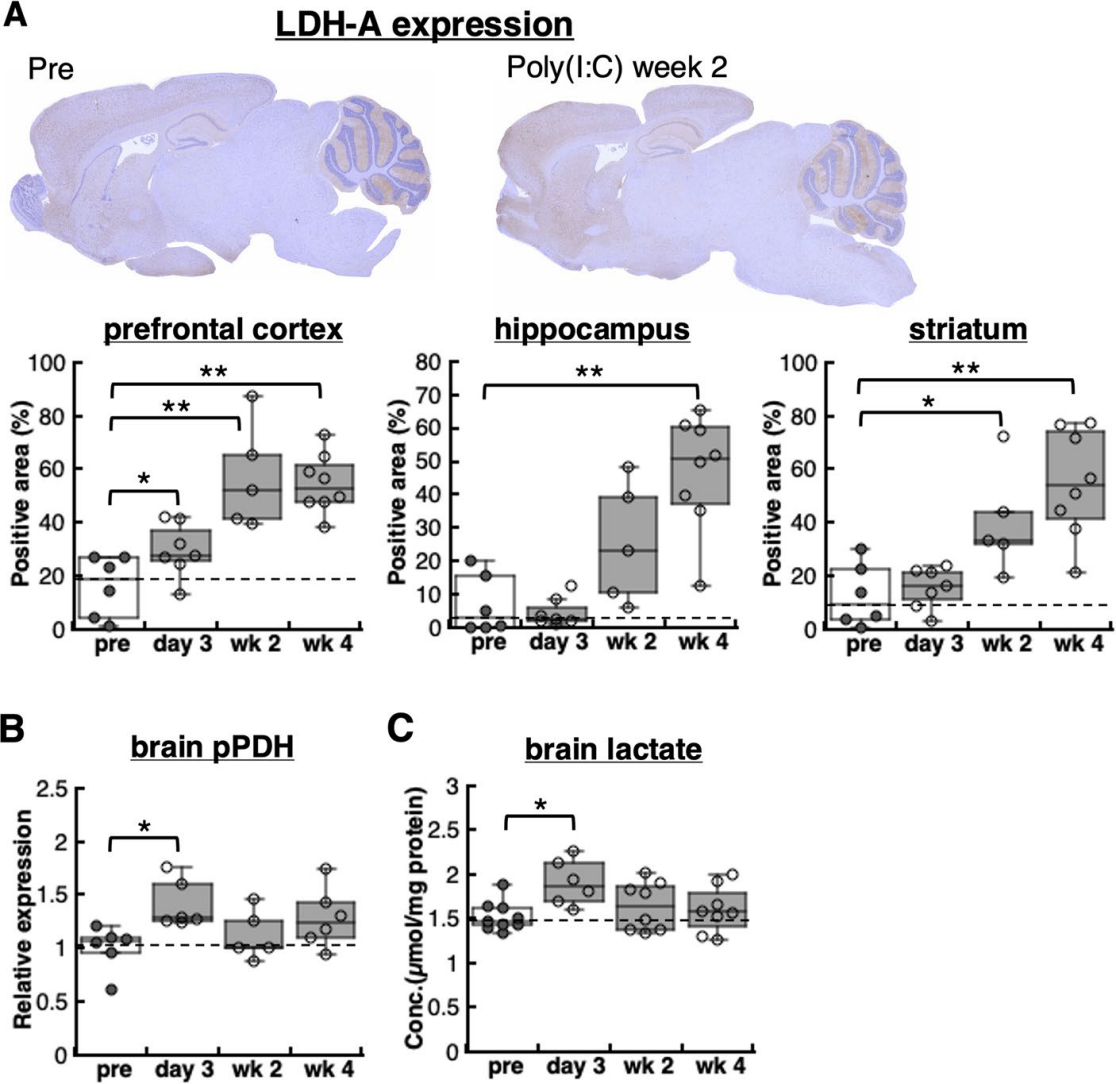
Sustained brain metabolic alteration in the pseudo-infection mouse model. **A** Immunohistochemical analysis showed that LDH-A expression levels significantly increased in the prefrontal cortex at 3 days, 2 weeks, and 4 weeks after the start of Poly(I: C) administration. In contrast, LDH-A expression in the hippocampus and striatum showed a delayed but sustained increase following Poly(I: C) administration (*n* = 4–5). **B** A simple western analysis revealed significantly increased phosphorylated PDH levels, indicating suppression of metabolic flux to oxidative phosphorylation in the brain of pseudo-infection model mice after 3 days of Poly(I: C) administration (*n* = 6–9). **C** Brain lactate concentrations significantly increased in the murine pseudo-infection model after 3 days of Poly(I: C) treatment and remained higher than pre-administration levels even 4 weeks later. *n* = 6–9, *p < 0.05, **p < 0.01. LDH-A, lactate dehydrogenase A; PDH, pyruvate dehydrogenase

To further investigate the time-course of brain energy metabolic alterations in the murine pseudo-infection model, an immunohistochemical analysis of lactate dehydrogenase A (LDH-A) expression was performed. LDH-A expression levels quickly increased in the prefrontal cortex after 3 days of Poly(I: C) treatment, whereas most other regions, including the hippocampus and striatum, exhibited a delayed and sustained response to Poly(I: C), with the maximum increase observed at 4 weeks post-treatment (Fig. 6a). A simple western analysis of the whole brain revealed that the phosphorylation of pyruvate dehydrogenase (PDH), a marker of suppressed metabolic flux to oxidative phosphorylation, significantly increased only during the acute phase of Poly(I: C) treatment (Fig. 6b). The brain lactate concentration also significantly increased after 3 days of Poly(I: C) treatment (Fig. 6c).

The observed suppression of oxidative phosphorylation flux during the acute phase of Poly(I: C) treatment may reflect a shift in energy metabolism associated with neuroinflammatory stress. Both systemic and central inflammatory responses can impair mitochondrial function, leading to a compensatory increase in glycolytic metabolism. In this study, this metabolic shift was accompanied by reduced dopamine signal markers in the striatum and midbrain regions, suggesting that dopaminergic neurons may be particularly vulnerable to Poly(I: C)-induced neuroinflammation, consistent with a previous report [18]. Given the high energy demands of dopamine synthesis and release, impaired oxidative metabolism could directly limit dopaminergic activity. Moreover, inflammatory cytokines such as TNF-α and IL-1β, which are upregulated following Poly(I: C) administration, have been reported to inhibit mitochondrial respiration and downregulate the expression of key components of the electron transport chain [37]. These factors may collectively contribute to the suppression of oxidative phosphorylation, attenuation of dopamine-related signalling, and the behavioural abnormalities observed in this study.

Considering the time-course of alterations in energy metabolic markers and hyperpolarised ^13^C MRI of pyruvate metabolism in the brain, the metabolic flux of glucose appears to be redirected to glycolysis due to the suppression of oxidative phosphorylation during the acute phase of Poly(I: C) treatment, whereas increased LDH-A expression may contribute to enhanced metabolic flux to lactate production during the late phase. Interestingly, the time-course of alterations in the Lac/Bic ratio observed in the hyperpolarised ^13^C MRI study mirrored the time-course of diminished spontaneous behaviour compared to that of dopamine signal marker levels. Each experiment was conducted using independent groups of mice to avoid potential confounding effects of hyperpolarised ^13^C MRI measurements—including exogenous pyruvate injection and stress induced by isoflurane anesthesia, physical restraint, and tail vein cannulation—on subsequent neurological, biochemical, and behavioral assessments. Future studies involving multiple measurements and the longitudinal monitoring of brain function and metabolic features in the same mouse are warranted to better understand the relationship between infection–induced functional abnormalities in the brain and metabolic alterations.

## Conclusion

This study highlights the utility of hyperpolarised ^13^C-labelled pyruvate MRI as a non-invasive and highly sensitive quantum imaging biomarker for detecting persistent brain metabolic alterations in virus-mimicking Poly(I: C)–induced pseudo-infection, a condition lacking discernible morphological abnormalities. By enabling the visualisation of metabolic shifts such as enhanced glycolytic flux, hyperpolarised ^13^C MRI provides unique insight into the underlying mechanisms of neurofunctional impairments induced by infection, immune fatigue, and neurological inflammation. These findings underscore its potential as a diagnostic tool for evaluating subtle brain dysfunctions in neurodegenerative and psychiatric disorders, particularly when conventional imaging techniques fail to detect significant structural changes.

## Data Availability

The datasets generated and/or analyzed during the current study are available from the corresponding author on reasonable request.
